# Amyloid-linked versus age-driven copathologies in Alzheimer’s dementia: differential associations with *APOE* ε4

**DOI:** 10.21203/rs.3.rs-9044264/v1

**Published:** 2026-03-16

**Authors:** Íñigo Rodríguez-Baz, Lidia Vaqué-Alcázar, Lucía Maure-Blesa, Lucía Pertierra, Javier Arranz Martinez, Laura Molina-Porcel, Iban Aldecoa, Naomi Ferreira, Regina Paradela, Laura Videla, Isabel Barroeta, María Carmona-Iragui, Ma Belén Sánchez-Saudinós, Judit Selma-González, Oriol Dols-Icardo, Mateus Rozalem Aranha, Sònia Sirisi Dolcet, Carla Abdelnour, Ignacio Illán-Gala, Daniel Alcolea, Alberto Lleo, Claudia Suemoto, Juan Fortea

**Affiliations:** Sant Pau Memory Unit, Department of Neurology, Hospital de la Santa Creu i Sant Pau, Institut de Recerca Sant Pau (IR SANT PAU); University of Barcelona; Biomedical Research Institute Sant Pau; Sant Pau Memory Unit, Department of Neurology, Hospital de la Santa Creu i Sant Pau, Institut de Recerca Sant Pau (IR SANT PAU); Sant Pau; Hospital Clínic Barcelona; cHospital Clínic Barcelona; University of Sao Paulo Medical School; University of Sao Paulo; Biomedical Research Institute Sant Pau; Biomedical Research Institute Sant Pau; Biomedical Research Institute Sant Pau; Sant Pau Memory Unit, Department of Neurology, Hospital de la Santa Creu i Sant Pau, Institut de Recerca Sant Pau (IR SANT PAU); Sant Pau Memory Unit, Department of Neurology, Hospital de la Santa Creu i Sant Pau, Institut de Recerca Sant Pau (IR SANT PAU); Institut de recerca de l’hospital de Sant Pau; Institut de Recerca Hospital de la Santa Creu i Sant Pau; Institut de Recerca Sant Pau (IR SANT PAU), Hospital de la Santa Creu i Sant Pau; Sant Pau Memory Unit, Department of Neurology, Hospital de la Santa Creu i Sant Pau, Institut de Recerca Sant Pau (IR SANT PAU); Sant Pau Memory Unit, Hospital de la Santa Creu i Sant Pau - Biomedical Research Institute Sant Pau- Universitat Autònoma de Barcelona; Hospital de Sant Pau - IIB Sant Pau; Institut de Recerca Sant Pau (IR SANT PAU), Hospital de la Santa Creu i Sant Pau; University of Sao Paulo; Sant Pau Memory Unit, Hospital de la Santa Creu i Sant Pau - Biomedical Research Institute Sant Pau-Universitat Autònoma de Barcelona

## Abstract

The mechanisms by which apolipoprotein E (*APOE*) drives copathologies in established Alzheimer’s disease (AD) dementia via amyloid-dependent versus age-driven pathways remain unresolved. Analyzing data from 11,897 autopsied individuals from the National Alzheimer’s Coordinating Center, with copathology analyses restricted to amyloid-positive AD dementia, we show that *APOE* effects followed two distinct trajectories. Cerebral amyloid angiopathy exhibited a striking ε4 dose-response (OR = 5.76, 95% CI: 4.20–7.96, p < 0.001; for ε4/ε4 compared to ε3/ε3), whereas arteriolosclerosis and atherosclerosis risk increased with age, independent of *APOE* haplotype. Lewy body pathology showed modest *APOE* associations restricted to limbic/amygdalar-predominant forms and was related to dementia duration, suggesting AD-mediated secondary synucleinopathy. TDP-43 pathology was associated with chronological age, demonstrating regional progression with minimal *APOE* dependence. These findings suggests that in amyloid-positive AD dementia, *APOE* ε4 selectively amplifies amyloid-related pathology, particularly cerebral amyloid angiopathy, while other copathologies accumulate through age-driven, *APOE* haplotype-independent processes.

## Introduction

Alzheimer’s disease (AD) is the most common cause of dementia, neuropathologically characterized by the accumulation of amyloid plaques and tau neurofibrillary tangles. However, previous studies have demonstrated that the majority of patients with dementia present other neuropathologies at autopsy, frequently encompassing Lewy body pathology, TAR DNA-binding protein 43 (TDP-43) proteinopathy, cerebral amyloid angiopathy (CAA), and various forms of cerebrovascular disease. These studies have traditionally examined copathologies in dementia patients as largely independent contributors, often interpreted in the context of aging^1,2^. However, evidence from genetically determined AD, both in Autosomal Dominant AD (ADAD)^3^ and Down syndrome-associated AD (DSAD)^4^, have shown that other neuropathological hallmarks beyond amyloid and tau are frequent despite the young age at which dementia manifests in these populations.

The apolipoprotein E (*APOE*) gene represents the strongest common genetic risk factor for sporadic AD. The three major allelic variants (ε2, ε3, and ε4) confer differential risk, with ε4 associated with increased susceptibility and ε2 with relative protection against AD^5,6^. Beyond its established role in amyloid metabolism, some studies have shown that carriers of the *APOE* ε4 allele have more α-synuclein ^7,8^ and TDP-43 pathologies^9^, as well as increased CAA burden^10^. Recent evidence suggests that *APOE* ε4 homozygosity represents a genetically determined form of AD with near-complete penetrance of AD biology^11^. However, despite the much higher prevalence and the earlier presentation of AD symptoms in *APOE* ε4/ε4, when analyzing AD patients at dementia stage with amyloid confirmation, there were no differences across haplotypes, but younger individuals had higher tau positron emission tomography (PET) uptake^11^. This has been confirmed in other studies that have shown age-associated increases in tau PET burden in cognitively unimpaired individuals but decreases with age in patients with AD dementia^12,13^.

Age is indeed a fundamental determinant of both neurodegenerative and vascular pathologies. Furthermore, the temporal evolution of copathologies in relation to dementia duration remains poorly understood. Whether *APOE* haplotype modifies the severity or distribution of Lewy body, TDP-43, and vascular pathologies in relation to dementia duration has not been systematically examined. Disentangling the relative contributions of *APOE* haplotype and chronological age in neuropathologically confirmed AD dementia patients to diverse neuropathological outcomes is therefore critical for understanding disease mechanisms.

The objectives of this study were to evaluate whether *APOE* haplotypes are associated with neuropathological copathologies in individuals with neuropathologically confirmed AD dementia, while accounting for the potential confounding and modifying effects of age and dementia duration. Specifically, we aimed to: (1) examine whether *APOE* haplotypes exhibit different burden and regional specificity in their associations with Lewy body and TDP-43 pathologies; and (2) determine the relative contributions of *APOE* haplotype, age, and dementia duration to the severity of CAA, arteriolosclerosis, and atherosclerosis. By addressing these questions, this study seeks to refine our understanding of the role of *APOE* in shaping the heterogeneous neuropathological landscape of AD, with implications for mechanistic understanding, risk stratification, and the design of future clinical trials and therapeutic development.

## Results

### 2.1 Sample characteristics and distribution of *APOE* haplotype

We analyzed neuropathological and clinical data from 11,897 autopsied individuals enrolled across National Institute on Aging-funded Alzheimer’s Disease Research Centers and aggregated in the National Alzheimer’s Coordinating Center (NACC) repository. Table 1 summarizes the demographic, clinical, and neuropathological features of the 11,897 individuals included in the study. The most common *APOE* haplotypes were ε3/ε3 (47.5%), followed by ε3/ε4 (33.4%) and ε4/ε4 (8.0%). Compared with ε3/ε3 carriers, both ε3/ε4 and ε4/ε4 carriers showed earlier ages at dementia diagnosis (median −1.7 and −5.6 years, respectively) and death (−2.2 and −6.7 years; all p < 0.001). Overall, 48.3% of the cohort were female and 89.4% were White. At the time of death, 73.1% had received a dementia diagnosis, and 57.6% had been clinically classified as having AD. Moderate or frequent neuritic plaques were present in 63.6% of participants.

Amyloid positivity rates varied markedly across haplotypes in the full cohort. Among participants with a clinical diagnosis of AD dementia, amyloid positivity (defined as moderate or frequent neuritic plaques) was present in 88.7% of ε4/ε4 carriers, 79.3% of ε3/ε4 carriers, and 68.5% of ε3/ε3 carriers (Supplementary Figure 1, Supplementary Table 1). Similarly, concordant clinicopathological AD diagnosis was significantly more common among ε3/ε4 and ε4/ε4 carriers compared to ε3/ε3 carriers (Odds ratio [OR] = 2.73, with 95% confidence interval [95% CI] = 2.41–3.10 and OR 5.59, 95% CI 4.46–7.07, respectively; both p<0.001; Supplementary Table 2).

To ensure analyses focused on neuropathologically confirmed AD and to avoid confounding by differential amyloid rates, subsequent copathology analyses (sections 2.2 and 2.3) were restricted to AD dementia cases with confirmed amyloid deposition. This restriction ensured that observed *APOE* effects on copathologies reflected associations within established AD pathology rather than differences in AD diagnostic accuracy across haplotypes. Of note, in both clinically diagnosed and amyloid-positive AD dementia, the prevalence of Braak stage V–VI decreased with increasing age at death, with the exception of ε4/ε4 carriers, who showed no appreciable age effect and consistently the highest prevalence of Braak stage V–VI across all age groups (Supplementary Figure 2, Supplementary Table 3).

### 2.2 Lewy body and TDP-43 pathologies

**Figure 1.**
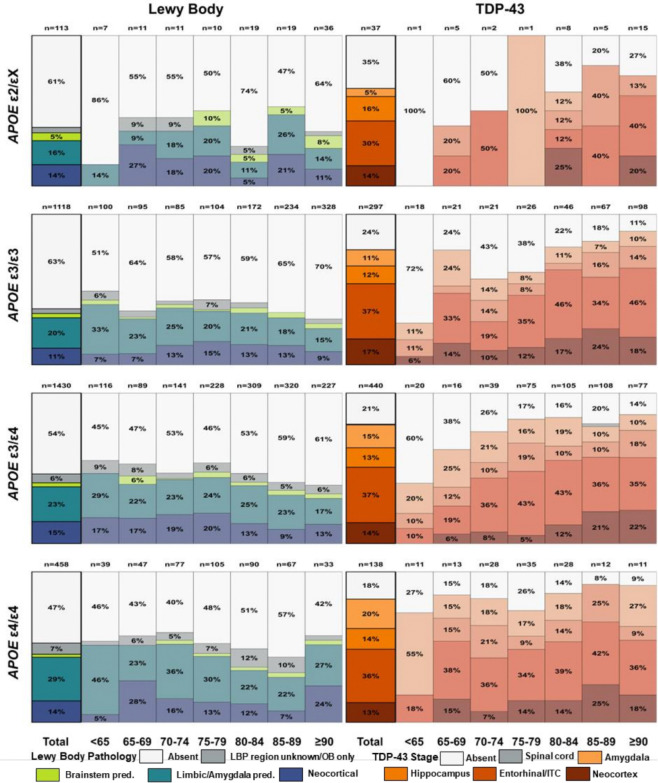
Distribution of Lewy body and TDP-43 pathology by age at death and APOE haplotype among individuals with amyloid-positive AD dementia. Stacked bar charts show, for ε2/X (top), ε3/ε3 (second), ε3/ε4 (third), and ε4/ε4 (bottom) carriers, the percentage of individuals with each pathology category across age-at-death groups. Left panels depict Lewy body pathology (Absent; LBP region unknown/OB only; Brainstem pred.; Limbic/Amygdala pred.; Neocortical) and right panels depict TDP-43 pathology (Absent; Spinal cord; Amygdala; Hippocampus; Entorhinal/lTC; Neocortical). Numbers above bars indicate the number of individuals in each age–haplotype stratum; thick black outlines highlight totals across all ages within each haplotype. AD, Alzheimer Disease; *APOE,* apolipoprotein E; ITC, Inferior Temporal Cortex; OB, Olfactory Bulb; pred., predominant; TDP-43, TAR DNA-binding protein 43.

Lewy body pathology was prevalent among individuals with amyloid-positive AD dementia, affecting more than one-third of ε3/ε3 carriers and over half of ε4/ε4 carriers overall (Figure 1, left panels). Prevalence increased with *APOE* ε4 dose, from 37.2% in ε3/ε3 to 46.2% in ε3/ε4 (OR = 1.39, 95% CI: 1.14–1.71, p = 0.001) and 52.6% in ε4/ε4 carriers (OR = 1.77, 95% CI: 1.33–2.36, p < 0.001). Across haplotypes, the most common regional pattern was limbic/amygdala-predominant involvement, followed by neocortical pathology, whereas brainstem-predominant Lewy pathology was comparatively infrequent.

Regional distributions differed modestly by *APOE* haplotype. Compared with ε3/ε3 carriers, ε3/ε4 carriers had higher prevalence of olfactory bulb/region-unknown pathology (5.9% vs 3.1%; OR = 2.27, 95% CI: 1.14–4.93, p = 0.018) and neocortical involvement (14.5% vs 11.0%; OR = 1.50, 95% CI: 1.11–2.04, p = 0.008). ε4/ε4 carriers showed greater limbic/amygdalar involvement than ε3/ε3 carriers (29.0% vs 20.2%; OR = 1.63, 95% CI: 1.21–2.19, p = 0.001). However, despite these differences, no consistent dose-dependent shift toward more extensive regional involvement was observed across ε4 allele groups (Supplementary Table 4A).

Lewy body pathology showed no significant association with age at death across *APOE* haplotypes (all likelihood ratio chi-squared tests [LRχ^2^] ≤ 2.54, p ≥ 0.111), consistent with the relatively stable prevalence across age strata shown in Figure 1. In regional analyses, increasing age was associated with lower amygdala involvement in ε3/ε3 carriers (OR per 10 years = 0.80, 95% CI: 0.68–0.93, p = 0.004), but no other significant age-related changes were observed in other regions or haplotypes (Supplementary Figure 3; Supplementary Table 5A).

**Figure 2.**
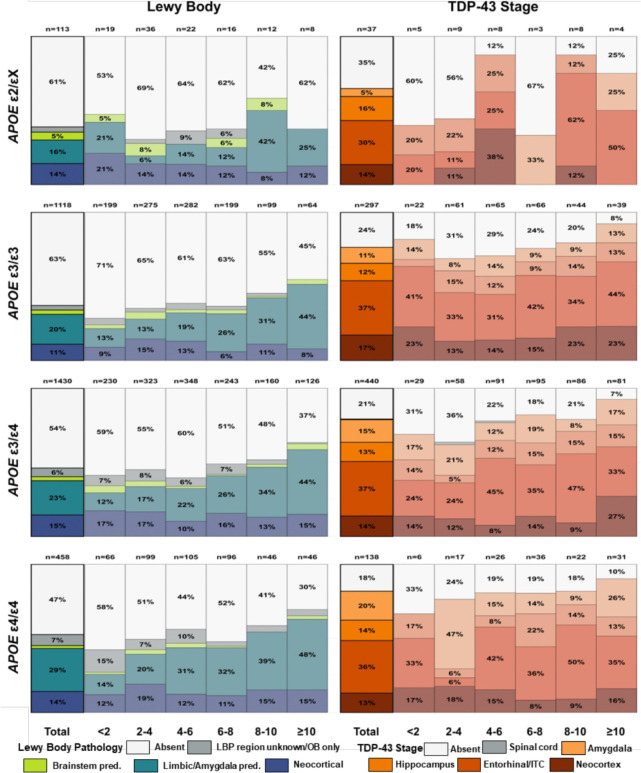
Distribution of Lewy body and TDP-43 pathology by dementia duration and *APOE* haplotype among individuals with amyloid-positive AD dementia. Stacked bar charts show, for ε2/X (top), ε3/ε3 (second), ε3/ε4 (third), and ε4/ε4 (bottom) carriers, the percentage of individuals with each pathology category across dementia-duration groups. Left panels depict Lewy body pathology (Absent; LBP region unknown/OB only; Brainstem pred.; Limbic/Amygdala pred.; Neocortical), and right panels depict TDP-43 pathology (Absent; Spinal cord; Amygdala; Hippocampus; Entorhinal/lTC; Neocortical). Numbers above bars indicate the number of individuals in each duration-haplotype stratum; thick black outlines highlight totals across all durations within each haplotype. AD, Alzheimer Disease; *APOE*, apolipoprotein E; ITC, Inferior Temporal Cortex; OB, Olfactory Bulb; pred., predominant; TDP-43, TAR DNA-binding protein 43.

In contrast, Lewy pathology was associated with dementia duration. Longer duration was linked to greater limbic/amygdalar involvement in ε3/ε3 and ε3/ε4 carriers after adjustment for sex, education, age at dementia onset and Alzheimer’s disease neuropathologic change (ADNC) classification (Figure 2, left panels; Supplementary Table 6A). This relationship was not observed in ε4/ε4 carriers, in whom (mainly limbic/amygdalar) Lewy pathology was already highly prevalent at shorter dementia durations, suggesting earlier or more rapid accumulation in this group.

TDP-43 pathology was even more prevalent than Lewy pathology across all *APOE* haplotypes (Figure 1, right panels). Overall prevalence exceeded three-quarters of individuals in each group, except ε2/εX, and was higher in ε4/ε4 compared with ε3/ε3 carriers (81.9% vs 76.4%; OR = 1.99, 95% CI: 1.16–3.50, p = 0.012). Across haplotypes, amygdala involvement was the dominant regional pattern, with hippocampal, entorhinal/inferior temporal, and neocortical involvement occurring in progressively smaller proportions.

Unlike Lewy pathology, TDP-43 showed a strong relationship with age at death. Overall prevalence increased with age in ε3/ε3 and ε3/ε4 carriers (both LRχ^2^ ≥ 10.74, p ≤ 0.001), accompanied by regional progression beyond the amygdala. Specifically, increasing age was associated with greater entorhinal/inferior temporal involvement in ε3/ε3 carriers (OR per 10 years = 1.51, 95% CI: 1.17–1.99, p = 0.002) and greater neocortical involvement in ε3/ε4 carriers (OR per 10 years = 2.01, 95% CI: 1.38–3.01, p < 0.001). Conversely, amygdala-only involvement declined with age in ε3/ε4 (OR per 10 years = 0.73, 95% CI: 0.55–0.99, p = 0.044) and ε4/ε4 carriers (OR per 10 years = 0.57, 95% CI: 0.31–0.98, p = 0.042) (Supplementary Figure 4; Supplementary Table 5B).

Despite its strong age dependency, TDP-43 pathology showed a weaker association with dementia duration. After adjustment for age at dementia onset, sex, education, and ADNC classification, longer duration remained associated with higher TDP-43 prevalence across most haplotypes (all LRχ^2^ ≥ 5.04, p ≤ 0.025), although not significantly in ε4/ε4 carriers (LRχ^2^ = 3.57, p = 0.059) (Figure 2, right panels). Regionally, longer duration was associated with increased entorhinal involvement in ε2/εX carriers and greater neocortical involvement in ε3/ε4 carriers, but these effects were modest compared with the age-related patterns (Supplementary Table 6B).

### 2.3 Vascular-related pathology

**Figure 3.**
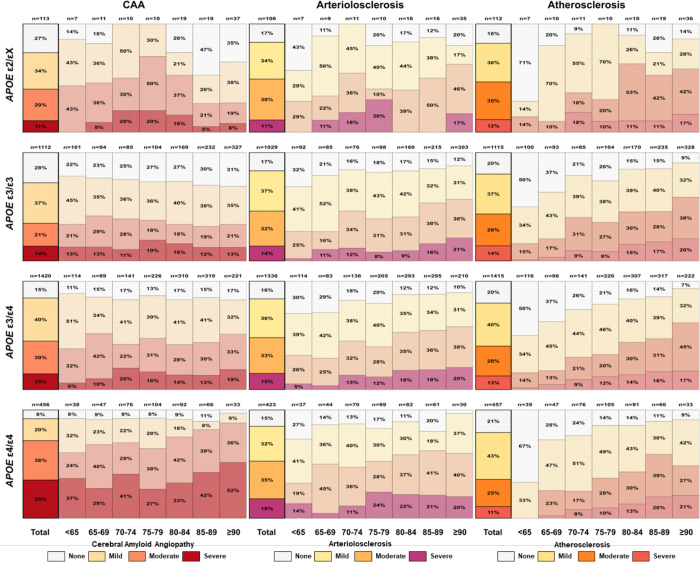
Distribution of cerebrovascular pathology severity by age at death and *APOE* haplotype among individuals with amyloid-positive AD dementia. Stacked bar charts show the percentage of ε2/X (top), ε3/ε3 (second), ε3/ε4 (third), and ε4/ε4 (bottom) carriers with none, mild, moderate, or severe pathology across age-at-death categories. Left panels depict cerebral amyloid angiopathy, middle panels cerebral arteriolosclerosis, and right panels atherosclerosis of the Circle of Willis. Numbers above each bar indicate the number of individuals in each age-haplotype stratum; thick black outlines highlight totals across all ages within each haplotype and pathology. AD, Alzheimer Disease; *APOE,* apolipoprotein E; CAA, Cerebral Amyloid Angiopathy.

CAA exhibited a striking *APOE* ε4 gene-dose effect, with increasing prevalence and severity across haplotypes. Compared with ε3/ε3 carriers, moderate-to-severe CAA was more common in ε3/ε4 carriers (OR = 1.54, 95% CI: 1.25–1.89, p < 0.001) and markedly elevated in ε4/ε4 carriers (OR = 5.76, 95% CI: 4.20–7.96, p < 0.001), with *APOE* ε4 homozygotes showing the highest proportions of both moderate and severe CAA (Supplementary Table 7A). This shift toward moderate and severe CAA was evident across all age strata, with ε4/ε4 carriers already showing a high burden at younger ages (Figure 3, left panels).

Age further modified CAA burden in ε4 carriers. Among ε4/ε4 individuals, CAA prevalence increased with advancing age (OR per 10 years = 1.69, 95% CI: 1.21–2.38, p = 0.002), a pattern also observed for severe CAA in ε3/ε4 carriers (Supplementary Table 8A; Supplementary Figure 5, upper panels). In contrast, age-related increases were less pronounced in ε3/ε3 carriers.

**Figure 4.**
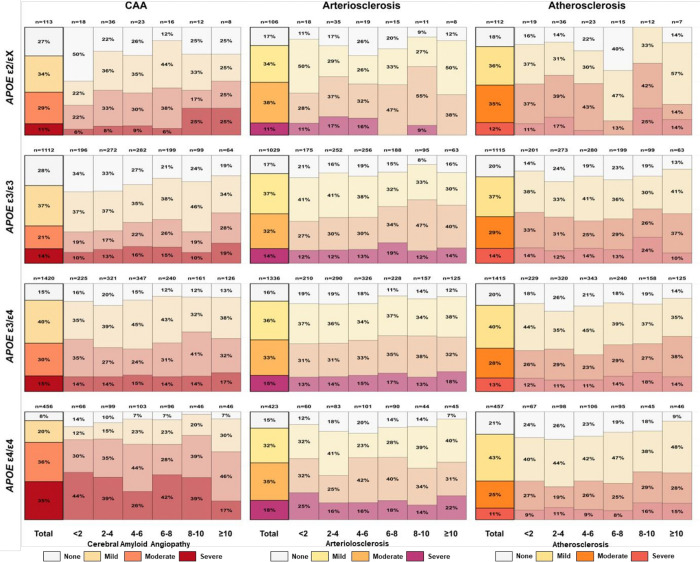
Distribution of cerebrovascular pathology severity by dementia duration and *APOE* haplotype among individuals with amyloid-positive AD dementia. Stacked bar charts show the percentage of ε2/X (top), ε3/ε4 (third), and ε4/ε4 (bottom) carriers with none, mild, moderate, or severe pathology across dementia-duration categories. Left panels depict cerebral amyloid angiopathy, middle panels cerebral arteriolosclerosis, and right panels atherosclerosis of the circle of Willis. Numbers above each bar indicate the number of individuals in each duration-haplotype stratum; thick black outlines highlight totals across all durations within each haplotype and pathology. AD, Alzheimer Disease; *APOE*, apolipoprotein E; CAA, Cerebral Amyloid angiopathy.

Dementia duration showed a distinct pattern. After full adjustment for age at dementia onset, sex, education, and ADNC classification, longer dementia duration was associated with increasing CAA prevalence in ε3/ε3 (OR per year = 1.09, 95% CI: 1.03–1.15, p = 0.002) and ε3/ε4 carriers (OR per year = 1.06, 95% CI: 1.02–1.11, p = 0.008; Figure 4, left panels). In ε4/ε4 carriers, however, longer duration was associated with lower prevalence of severe CAA (OR per year = 0.91, 95% CI: 0.84–0.98, p = 0.016), suggesting that severe CAA accumulates early in this haplotype (Supplementary Table 9A) and might be associated with survival.

In contrast to CAA, cerebral arteriolosclerosis and atherosclerosis of the circle of Willis showed weaker associations with *APOE* haplotype and were driven primarily by age (Figure 3, middle and right panels). Arteriolosclerosis prevalence was modestly higher in ε4/ε4 compared with ε3/ε3 carriers (52.7% vs 46.0%; OR = 1.49, 95% CI: 1.10–2.01, p = 0.009), driven mainly by severe pathology (OR = 1.69, 95% CI: 1.12–2.55, p = 0.013). By contrast, moderate-to-severe atherosclerosis prevalence did not differ significantly across *APOE* haplotypes, although ε4/ε4 carriers showed higher rates of mild atherosclerosis (Supplementary Table 7B-C).

Age was the dominant determinant of cerebral arteriolosclerosis and atherosclerosis of the circle of Willis. Prevalence of arteriolosclerosis increased with age across all haplotypes (all LRχ^2^ ≥ 5.96, p ≤ 0.015), with parallel increases in moderate and severe strata (Supplementary Table 8B). Similarly, atherosclerosis prevalence increased with age in all haplotypes (all LRχ^2^ ≥ 7.79, p ≤ 0.005), accompanied by progressive shifts toward greater severity (Supplementary Figure 5, middle and bottom panels).

Associations with dementia duration were comparatively modest. In fully adjusted models, arteriolosclerosis prevalence increased with longer disease duration only in ε3/ε3 carriers (LRχ^2^ = 18.84, p < 0.001), driven mainly by moderate pathology (Figure 4, middle panels; Supplementary Table 9B). Atherosclerosis prevalence increased with longer dementia duration in all haplotypes except ε2/εX (all LRχ^2^ ≥ 9.04, p ≤ 0.003), although the magnitude of these effects was smaller than those observed with age (Figure 4, right panels; Supplementary Table 9C).

## Discussion

In this large autopsy-based cohort of AD dementia with confirmed amyloid positivity, we found that *APOE* haplotype related to copathologies in a highly selective manner following two broad trajectories: amyloid-linked/*APOE*-dependent versus age-driven/*APOE*-independent. Lewy body and TDP-43 pathologies were both extremely frequent, but they diverged in their determinants: Lewy pathology showed modest *APOE*-related differences concentrated in limbic/amygdalar regions and was largely age-independent, yet it increased with dementia duration, positioning it as an “intermediate” process that may accrue alongside AD progression. TDP-43, in contrast, was less *APOE*-dependent and tracked primarily with age, with evidence of regional progression and comparatively weaker links to dementia duration. The vascular findings then provided the clearest dissociation: CAA was strongly ε4 dose-dependent, whereas non-CAA vascular disease (arteriolosclerosis and large-vessel atherosclerosis) was predominantly age-driven with limited haplotype effects. Notably, dementia duration related more to amyloid-linked pathology (CAA, and to some extent Lewy pathology) than to non-CAA vascular disease, reinforcing the interpretation that *APOE* ε4 chiefly amplifies amyloid-related mechanisms once dementia is established, while other copathologies accumulate largely through aging and disease-stage processes that are relatively *APOE* haplotype-independent.

*APOE* strongly shapes core AD pathology even in dementia-stage disease. *APOE* ε4/ε4 carriers were enriched for clinicopathological AD, consistent with near-complete penetrance of AD biology^11^. Furthermore, the age-invariant frequency of Braak V-VI in ε4/ε4 suggests early tau saturation, in line with tau-PET evidence^12,13,24^. Conversely *APOE* ε2 confers protection to AD pathology even in clinical AD dementia cases^5^. To reduce sample-composition bias when assessing copathologies, we focused our primary analyses on AD dementia with neuritic plaque-confirmed amyloid positivity, ensuring *APOE* effects were evaluated within established AD biology.

Lewy body pathology was more frequent overall in ε3/ε4 and ε4/ε4 carriers. However, the most informative signal lied in the topography. The only consistent *APOE*-related pattern was limbic/amygdalar involvement, which was higher in ε4/ε4 than ε3/ε3 carriers (29.0% vs 20.2%), whereas associations in olfactory and neocortical regions were less consistent. This topographic selectivity places Lewy pathology in an “intermediate” position between the amyloid-linked and age-driven axes: it showed modest haplotype effects concentrated in a vulnerable region, with no robust (or decreasing) age gradient. Importantly, there was an amydgalar accumulation (but not neocortical) Lewy pathology with dementia duration.

The concordant effects of *APOE* and dementia duration support the view that amygdala-predominant Lewy pathology often represents secondary, AD-mediated α-synucleinopathy rather than primary synuclein disease^25^. Notably, this amygdala-centered pattern is consistent with observations in genetically determined AD, including autosomal-dominant AD (ADAD) and Down syndrome-associated AD (DSAD), where Lewy pathology, when present, also shows a preferential limbic/amygdalar accumulation in the context of established AD biology ^26,27^. By contrast, the lack of a consistent *APOE* dose-response for neocortical (diffuse) Lewy pathology is compatible with a distinct pathological trajectory more closely linked to the Lewy body dementia phenotype and a more aggressive course that can be relatively independent of AD burden^28^.

TDP-43 pathology, by comparison, mapped much more cleanly onto an age-driven process. While ε4/ε4 carriers had slightly higher prevalence than ε3/ε3, we did not observe a significant ε3/ε4 effect within this amyloid-positive AD dementia cohort, differing from reports of ε4 dose-dependent associations in broader samples^9,29^. This discrepancy is plausibly explained by conditioning on established AD dementia with confirmed amyloidosis, which reduces heterogeneity but can also attenuate haplotype contrasts when baseline TDP-43 burden is already high. Consistent with this, ε3/ε4 effects on TDP-43 pathology emerged when we evaluated all-cause and unconfirmed-amyloid dementia groups (Supplementary Table 11). The dominant signals were again topographic and temporal: TDP-43 increased with age across most haplotypes and showed a shift from amygdala-predominant involvement toward broader limbic and neocortical spread, with dementia duration contributing more modestly than age. This pattern closely matches the defining clinical-pathological trajectory of Limbic-predominant age-related TDP-43 encephalopathy (LATE)^30^, supporting the interpretation that in amyloid-positive AD dementia, TDP-43 accumulation is driven primarily by aging and progressive regional spread, rather than by *APOE* haplotype per se. This age-driven, *APOE*-independent profile is further supported by genetic evidence showing that TDP-43 accumulation in aging brains follows a distinct genetic risk architecture: TMEM106B and GRN variants associate with TDP-43 pathology and hippocampal sclerosis independently of *APOE* haplotype^31^, and TNIP1, recently identified as a risk factor for frontotemporal lobar degeneration with TDP-43 pathology^32^, has been proposed to explain the TNIP1-AD genetic overlap through comorbid TDP-43 rather than amyloid mechanisms^33^.

The amygdala’s broader vulnerability to misfolded protein accumulation across disorders provides a plausible substrate for this convergence^34^. One parsimonious explanation is that the amygdala, together with other phylogenetically older cortices, acts as an early “landing zone” for multiple age- and AD-linked proteinopathies, with stereotyped staging schemes often beginning in limbic structures (e.g., LATE/TDP-43) before extending cortically^30^. In AD specifically, previous works had shown that α-synuclein deposition frequently shows an amygdala-predominant pattern, suggesting region-specific susceptibility rather than uniform neocortical spread^35^. Mechanistically, this convergence could reflect a combination of selective neuronal/architectural vulnerability and **interaction between pathologies,** including cross-seeding or permissive proteostatic/inflammatory milieus generated by AD pathology, supported by experimental work demonstrating tau-α-synuclein cross-seeding and prion-like propagation^36^. Consistent with this, AD cases with amygdala-predominant α-synuclein have been reported to show particularly high co-occurring TDP-43 burden and even co-localization of aggregates within limbic neurons, reinforcing the idea of a limbic “co-proteinopathy hub” rather than independent parallel processes^37^.

The strong *APOE*-related gradient we observed for CAA is well aligned with prior neuropathology literature showing that *APOE* ε4 preferentially amplifies amyloid deposition in the vessel wall^10^. In our amyloid-positive AD dementia cohort, haplotype outweighed both age and dementia duration as a correlate of CAA burden: ε4/ε4 carriers had an almost six-fold higher odds of moderate-to-severe CAA compared with ε3/ε3, and severity shifted upward with *APOE* ε4 gene dose. This pattern mirrors observations in clinicopathologically confirmed AD in which ε4 is more tightly coupled to severe CAA than to chronological age or disease duration^38^. The contrasting duration associations, positive in ε3/ε3 and ε3/ε4 but inverse for severe CAA in ε4/ε4, suggest that CAA is tightly coupled to AD biology; the ε4/ε4 pattern may be best explained by earlier attainment of severe CAA and consequent selection among longer survivors, as CAA is associated with increased mortality risk^39^.

In contrast, non-CAA vascular lesions aligned predominantly with an age-driven trajectory, consistent with the progressive accumulation of cerebrovascular risk factors not captured in our analyses, with substantially weaker *APOE* effects than those seen for CAA. Cerebral arteriolosclerosis showed at most a modest enrichment in ε4/ε4 carriers relative to ε3/ε3, but the dominant signal across haplotypes was a progressive shift toward greater severity with advancing age. Differences from population-based reports that observed weaker or null *APOE* associations^40^ may reflect both cohort composition (our restriction to neuropathologically confirmed AD dementia with amyloid positivity) and analytic choices (separating ε4 homozygotes rather than combining all ε4 carriers). Circle of Willis atherosclerosis similarly showed minimal haplotype-related differences, yet increased steadily with age across haplotypes, again aligning with prior neuropathological and cardiovascular pathology reports^40,41^. The additional, smaller association with dementia duration observed for atherosclerosis in our AD dementia sample may reflect cumulative exposure to systemic vascular risk and comorbidity during the clinical course, rather than an AD-specific genetic effect. Overall, these results sharpen a key distinction within amyloid-positive AD dementia: CAA behaves as an *APOE*/amyloid-linked vasculopathy, whereas arteriolosclerosis and large-vessel atherosclerosis largely reflect age- and risk-related cerebrovascular injury, with only limited modulation by *APOE* haplotype.

This study has important strengths and some limitations that inform future research. The large autopsy-confirmed sample and standardized neuropathological assessments provide exceptional statistical power to detect *APOE* haplotype-specific effects across a broad spectrum of copathologies and reduce the diagnostic uncertainty inherent in purely clinical or biomarker-based cohorts. However, the cohort lacks diversity, being predominantly White, which limits generalizability to population-based and ancestrally diverse samples. In addition, the cross-sectional nature of autopsy data and evolving NACC collection protocols, including inter-site variability in pathological ratings, constrain inferences about temporal trajectories of pathology despite the use of harmonized criteria. Additionally, cerebrovascular risk factors such as hypertension, diabetes, or dyslipidemia were not included as covariates in our analyses, which may partially account for the age-driven vascular pathology patterns observed and should be addressed in future studies. Furthermore, our focus on amyloid-positive AD dementia, while ensuring diagnostic homogeneity, precludes assessment of *APOE* effects in amyloid-negative dementia or preclinical stages, where associations may differ. However, as shown in the supplementary material, the NACC database is not a population-based cohort and is highly enriched for individuals with dementia. Therefore, it might not be well-suited to address these questions. These considerations underscore the need for longitudinal, multimodal studies with serial fluid and imaging biomarkers as well as postmortem validation in more diverse and population-based cohorts.

In conclusion, these data support a parsimonious model in which copathologies in amyloid-positive AD dementia distribute across distinct but overlapping axes. One axis is *APOE*/amyloid-linked, dominated by CAA severity and accompanied by selective limbic vulnerability, where Lewy pathology shows its most consistent (albeit modest) *APOE* signal. The other axis is age-driven and relatively *APOE*-independent, encompassing non-CAA vascular disease and TDP-43 pathology. This framework has practical implications for interpreting clinicopathologic variability and suggests that, in late-stage disease, trial stratification may benefit most from capturing amyloid-related vascular vulnerability and limbic-predominant co-proteinopathy patterns, rather than assuming broad *APOE* effects across all copathologies.

## Methods

### 4.1 Study cohort

We analyzed data from the March 2025 freeze of the NACC repository (https://www.naccdata.org/), which aggregates de-identified longitudinal clinical and neuropathological data from National Institute on Aging–funded Alzheimer’s Disease Research Centers (ADRCs). ADRCs enroll participants across the cognitive spectrum, from cognitively unimpaired individuals to those with dementia, although most participants have AD dementia and are typically recruited after symptom onset.

All contributing ADRCs obtained local institutional review board approval and written informed consent from participants or their legal representatives, and procedures adhered to the Declaration of Helsinki. NACC maintains a harmonized clinical database based on the Uniform Data Set (UDS) and a complementary neuropathology database based on standardized Neuropathology Forms. For this study, we included participants who had died and undergone brain autopsy and excluded individuals without *APOE* genotyping or carrying a known pathogenic variant associated with familial AD (NPPDXP), frontotemporal dementia (NPPDXQ), or Down syndrome (NACCDOWN).

### 4.2 Demographic, cognitive, and genetic variables

We extracted standard demographic variables from NACC. Sex (SEX) was coded as male or female. Race (RACE) was classified as White, Black or African American, American Indian or Alaska Native, Native Hawaiian or Other Pacific Islander, Asian, Other, or Unknown. Educational attainment was quantified as total years of formal education (EDUC). *APOE* haplotype was derived from the variable NACCAPOE and categorized as ε2/ε2, ε2/ε3, ε3/ε3, ε2/ε4, ε3/ε4, or ε4/ε4, with ε3/ε3 used as the reference group in all comparative analyses, given that it is the most common haplotype in the general population. Ages at diagnosis of mild cognitive impairment (MCI), dementia, and at death were calculated by combining the date of the visit at which MCI or dementia was first diagnosed (year, month, and day) with the recorded date of birth and date of death. Participants were then classified according to their last cognitive status as having dementia, MCI, or cognitively unimpaired (CU).

Within the UDS, the Etiologic Diagnoses section allows clinicians to indicate, for each potential cause of cognitive impairment, whether it is the primary etiology, a contributing factor, or not contributing; each category is coded as present or absent based on clinical judgment and available diagnostic information. For this study, we focused on whether AD was recorded as the primary etiologic diagnosis of cognitive impairment. Participants were therefore classified at the visit closest to death as having clinical AD (AD as the primary etiology) or clinical non-AD (a non-AD primary etiology).

### 4.3 Neuropathological variables

Neuropathological data were obtained from NACC Neuropathology Forms (versions 10–11) completed after autopsy^14^. ADNC was obtained from variable NPADNC, which integrates three components into a composite score (Not, Low, Intermediate, or High): Thal phase for amyloid plaque distribution (A score)^15^, Braak stage for neurofibrillary tangle burden (B score)^16^, and the Consortium to Establish a Registry for Alzheimer’s Disease (CERAD) neuritic plaque density (C score)^17^. We used CERAD semi-quantitative assessments of neuritic plaque burden (NACCNEUR: No, Sparse, Moderate, or Frequent neuritic plaques) and Braak stage for neurofibrillary tangle burden (NACCBRAA: Stage 0–VI; recoded as None or stages I–II, III–IV, V–VI). Given the central role of amyloid biomarkers in research and clinical practice, CERAD neuritic plaque severity was collapsed into a binary amyloid status: individuals with “No” or “Sparse” neuritic plaques were considered amyloid-negative, and those with “Moderate” or “Frequent” plaques as amyloid-positive. This dichotomization was chosen to approximate amyloid PET positivity and align with current AD neuropathologic change frameworks^18–20^.

Lewy body pathology was assessed using the NACC-derived variable NACCLEWY, which classifies cases as: no Lewy body pathology, brainstem-predominant, limbic or amygdala-predominant, neocortical, or Lewy bodies present in unspecified region/olfactory bulb. These categories are broadly consistent with the DLB Consortium neuropathological classification framework^21^, though NACC combines limbic and amygdala-predominant patterns into a single category. TDP-43 immunoreactive inclusions were assessed across five brain regions, spinal cord (NPTDPA), amygdala (NPTDPB), hippocampus (NPTDPC), entorhinal/inferior temporal cortex (NPTDPD), and neocortex (NPTDPE), as presence or absence; regional distribution patterns are consistent with LATE-NC staging criteria^22,23^. Cerebrovascular pathologies were graded using NACC semi-quantitative scales (none, mild, moderate, severe), including cerebral amyloid angiopathy (NACCAMY), arteriolosclerosis (NACCARTE), and Circle of Willis atherosclerosis (NACCAVAS)^14^.

### 4.5 Statistical analyses

Categorical variables were summarized as counts and percentages by *APOE* haplotype (i.e., ε2/εX, ε3/ε3, ε3/ε4, ε4/ε4), and continuous variables were reported as medians with interquartile ranges. To examine associations between *APOE* haplotype and key clinical and pathological outcomes at death, we fitted binary logistic regression models using *APOE* ε3/ε3 as the reference category.

Given the low representation of *APOE* ε2/ε2 (n = 44) and ε2/ε3 (n = 948), these groups were combined into a single category (ε2/εX) for neuropathological analyses to ensure adequate statistical power. Individuals with the ε2/ε4 haplotype (n = 331) were excluded due to the opposing effects of *APOE* ε2 and ε4 alleles on AD risk and the small sample size.

Neuropathological outcomes were analyzed using regression models. For binary outcomes (presence or absence of pathology), we applied logistic regression. For ordered severity measures (Lewy body pathology distribution, TDP-43 pathology stage, and vascular pathology severity grades), we used proportional odds ordinal logistic regression. Models were fitted unadjusted and then progressively adjusted, first for sex and years of education, then additionally for age at death or age at dementia diagnosis as appropriate, and finally with the addition of AD neuropathologic change (ADNC) classification. ORs with 95% CIs were estimated from these models. Global effects of *APOE* haplotype, age at death, and dementia duration were assessed using LRχ^2^. Given that progressive model adjustments represent sequential refinements of the same associations rather than independent tests, we did not apply corrections for multiple comparisons. Interpretation emphasized both effect magnitude (ORs with 95% CIs) and statistical significance.

Analyses of Lewy body pathology, TDP-43 pathology, CAA, arteriolosclerosis, and Circle of Willis atherosclerosis were restricted to participants with a clinical diagnosis of AD dementia and neuropathologically confirmed amyloid positivity (moderate or frequent neuritic plaques by CERAD criteria). Age-related analyses used the following age-at-death categories: <65, 65–69, 70–74, 75–79, 80–84, 85–89, ≥90 years. Dementia duration was categorized as <2, 2–3, 4–6, 6–8, and ≥10 years to capture clinically distinct phases of disease progression while maintaining sufficient cases per group. Distributions of neuropathological features across these categories were assessed using chi-squared tests (χ^2^).

Additional analyses are described in the Supplementary Methodology, including: (1) cohort characterization and rationale for restricting analyses to amyloid-positive AD dementia cases (Supplementary Figure 1, Supplementary Tables 1A-B and 2); (2) Braak neurofibrillary tangle staging across multiple diagnostic groups (all-cause dementia, AD dementia, and amyloid-positive AD dementia) (Supplementary Figure 2, Supplementary Tables 3 and 4); (3) *APOE* × age interaction testing for regional Lewy body, TDP-43, and cerebrovascular pathologies (Supplementary Figures 3, 4 and 5), (4) sex-stratified sensitivity analyses for all neuropathologies (Supplementary Table 10), and (5) TDP-43 pathology across diagnostic groups (Supplementary Table 11).

All statistical analyses were performed using R (version 4.2.2). Tests were two-sided, and P < 0.05 was considered statistically significant.

## Supplementary Material

Table 1 is available in the Supplementary Files section.

This is a list of supplementary files associated with this preprint. Click to download.

- SUPPLEMENTARYMATERIALAPOEcopathology.docx
- Table1.docx

## Data Availability

The data that support the findings of this study are available from the National Alzheimer’s Coordinating Center (NACC; https://www.naccdata.org/) upon registration and approval.
